# Regulation of 20α-Hydroxysteroid Dehydrogenase Expression in Term Pregnant Human Myometrium Ex Vivo

**DOI:** 10.1007/s43032-023-01333-6

**Published:** 2023-08-30

**Authors:** Marina Paul, Anna Paredes Barreda, Amy Gregson, Richard Kahl, Madeline King, Waleed M. Hussein, Frederick R. Walker, Roger Smith, Tamas Zakar, Jonathan W. Paul

**Affiliations:** 1 School of Biomedical Sciences and Pharmacy, College of Health, Medicine and Wellbeing, University of Newcastle, Callaghan, NSW 2308 Australia; 2 Hunter Medical Research Institute, New Lambton Heights, NSW 2305 Australia; 3 Centre for Rehab Innovations, University of Newcastle, Callaghan, NSW 2308 Australia; 4 School of Medicine and Public Health, College of Health, Medicine and Wellbeing, University of Newcastle, Callaghan, NSW 2308 Australia; 5 Mothers and Babies Research Program, New Lambton Heights, NSW 2305 Australia; 6 Institute for Molecular Bioscience, The University of Queensland, Brisbane, St. Lucia, QLD 4072 Australia; 7 John Hunter Hospital, New Lambton Heights, NSW 2305 Australia

**Keywords:** Myometrium, Progesterone, *AKR1C1*, 20α-HSD, Histone deacetylase inhibitors, Trichostatin A

## Abstract

Metabolic inactivation of progesterone within uterine myocytes by 20α-hydroxysteroid dehydrogenase (20α-HSD) has been postulated as a mechanism contributing to functional progesterone withdrawal at term. In humans, 20α-HSD is encoded by the gene *AKR1C1*. Myometrial *AKR1C1* mRNA abundance has been reported to increase significantly during labor at term. In spontaneous preterm labor, however, we previously found no increase in *AKR1C1* mRNA level in the myometrium except for preterm labor associated with clinical chorioamnionitis. This suggests that increased 20α-HSD activity is a mechanism through which inflammation drives progesterone withdrawal in preterm labor. In this study, we have determined the effects of various treatments of therapeutic relevance on *AKR1C1* expression in pregnant human myometrium in an ex vivo culture system. *AKR1C1* expression increased spontaneously during 48 h culture (*p* < 0.0001), consistent with the myometrium transitioning to a labor-like phenotype ex vivo*,* as reported previously. Serum supplementation, prostaglandin F_2α_, phorbol myristate acetate, and mechanical stretch had no effect on the culture-induced increase, whereas progesterone (*p* = 0.0058) and cAMP (*p* = 0.0202) further upregulated *AKR1C1* expression. In contrast, culture-induced upregulation of *AKR1C1* expression was dose-dependently repressed by three histone/protein deacetylase inhibitors: trichostatin A at 5 (*p* = 0.0172) and 25 µM (*p* = 0.0115); suberoylanilide hydroxamic acid at 0.5 (*p* = 0.0070), 1 (*p* = 0.0045), 2.5 (*p* = 0.0181), 5 (*p* = 0.0066) and 25 µM (*p* = 0.0014); and suberoyl bis-hydroxamic acid at 5 (*p* = 0.0480) and 25 µM (*p* = 0.0238). We propose the inhibition of histone/protein deacetylation helps to maintain the anti-inflammatory, pro-quiescence signaling of progesterone in pregnant human myometrium by blocking its metabolic inactivation. Histone deacetylase inhibitors may represent a class of agents that preserve or restore the progesterone sensitivity of the pregnant uterus.

## Introduction

The steroid hormone progesterone (P4) is essential for establishing and maintaining pregnancy [1–3]. The withdrawal of P4 action signals the end of pregnancy and in most mammalian species this occurs through a rapid fall in circulating levels of P4, which precipitates labor [4–8]. However, in humans and higher primates, circulating P4 levels remain elevated up to and during labor [9–11]. Nonetheless, blocking the actions of P4 by progesterone antagonists (e.g., RU486) promotes cervical ripening and labor in humans [12, 13]. This indicates that P4 action is essential in maintaining human pregnancy and suggests that P4 withdrawal at parturition occurs functionally rather than due to a systemic decline in the hormone level. In 2016, Nadeem et al*.* [14] provided evidence that functional P4 withdrawal results from the combination of signaling changes mediated by the nuclear P4 receptors, PR-A and PR-B, and the increased metabolic inactivation of P4 in the uterine tissue. During pregnancy, P4-liganded PR-B associates with Jun/Jun homodimers and the repressor complex, P54nrb/Sin3A/HDAC, to repress the transcription of *GJA1*, which encodes the key contraction-associated protein (CAP), connexin-43 [14]. During labor, PR-A, the truncated PR isoform, dissociates from P4 and interacts with Fos/Jun heterodimers, which activates the transcription of *GJA1*, thereby promoting term labor [14]. The reduction of P4 abundance and the appearance of unliganded PR-A in the myocyte nuclei were associated with the increased expression of the P4 metabolizing enzyme, 20α-hydroxysteroid dehydrogenase (20α-HSD), within uterine myocytes.

20α-HSD, a member of the aldo–keto reductase (AKR) superfamily, catalyzes the conversion of P4 to its inactive metabolite, 20α-hydroxyprogesterone (20α-OHP) [15]. The enzyme is encoded by the *AKR1C1* gene, which, in humans, is located on chromosome 10p15-p14. Prior research has shown that *AKR1C1* mRNA abundance and 20α-HSD protein levels significantly increased in human myometrium during term labor [14, 16]. Recently, Nadeem et al. [17] found upregulation of *AKR1C1* during labor in human and mouse myometrium as well as in mouse models of preterm labor (lipopolysaccharide (LPS)- and RU486-model). Furthermore, mechanistic studies with immortalized cells have shown that nuclear factor kappa-light-chain-enhancer of activated B cells (NF-κB) / activator protein 1 (AP-1) transcription factors mediated effects of the pro-inflammatory mediators, LPS and 12-*O*-tetradecanoylphorbol-13-acetate (TPA), respectively, on 20α-HSD gene transcription [17]. The increase in 20α-HSD led to local P4 withdrawal, which was concomitant with the upregulated transcription of CAPs, especially *GJA1*, thereby promoting myometrial cell contractility and labor [17]. In our recent study of pregnant human myometrium, we confirmed that myometrial *AKR1C1* expression (mRNA abundance) was upregulated during labor onset at term [18]. We also examined *AKR1C1* expression within preterm human myometrium but found no increase in *AKR1C1* mRNA abundance in association with preterm labor [18]. *AKR1C1* expression was, however, significantly upregulated in the myometrium of women who were in preterm labor and had clinical chorioamnionitis, suggesting that increased *AKR1C1* expression and 20α-HSD activity may be a mechanism through which inflammation drives progesterone withdrawal in preterm labor [18]. Taken together, these findings highlight the involvement of 20α-HSD in the timing of parturition and indicate a link between uterine inflammation and local P4 withdrawal that triggers labor. It is therefore of paramount importance to understand how 20α-HSD is regulated in human myometrium.

In the present study, we determined the effects of hormones, mechanical stretch, and pharmacologic treatments on myometrial *AKR1C1* expression utilizing our ‘ex vivo labor model’ of the human myometrium [19, 20]. We developed this model by culturing non-laboring term pregnant human myometrial tissue samples for 48 h, during which time the expression of the contraction-associated genes, *ESR1*, *PTGS2, OXTR,* and *PGR*, changed in a manner that is consistent with the transition to a pro-contractile, labor-like phenotype [19, 20]. We have demonstrated previously that treatments with steroid hormones or activators or inhibitors of signaling pathways modify contraction-associated gene expression in this ex vivo labor model [19, 20]. Notably, trichostatin A (TSA), a histone deacetylase (HDAC) inhibitor (HDACi), repressed the spontaneous upregulation of *PR-A* while having no effect on *PR-B* expression, thus maintaining a low *PR-A/PR-B* expression ratio that is characteristic of non-laboring myometrium and believed to be consistent with the maintenance of progesterone responsiveness [20]. Here, we show that *AKR1C1* mRNA levels increase spontaneously in our ex vivo labor model, consistent with other contraction-associated genes, and that three HDACis, TSA, suberoylanilide hydroxamic acid (SAHA), and suberoyl bis-hydroxamic acid (SBHA), repress the upregulation of *AKR1C1* expression, potentially blocking the metabolic arm of functional progesterone withdrawal.

## Materials and Methods

### Myometrial Tissue Acquisition

The study was approved by the Hunter and New England Area Human Research Ethics Committee (2019/ETH12330). Human myometrial samples were obtained after informed written consent of the donors from the upper lip of the incision in the lower uterine segment during cesarean section (CS) of singleton term pregnancies (38.3 – 39.5 weeks gestation). The patient body mass index (BMI) range was 20.3 – 33.6, and none of the patients was in labor. The indications for elective CS were previous CS, placenta previa, fetal distress, or breech presentation. Women were excluded if they were given steroids prenatally. Myometrial samples were placed on ice in a serum-free Dulbecco's Modified Eagle Medium (DMEM) with high glucose, 2 mM L-Glutamine, 1 mM Sodium Pyruvate, 40 µg/mL Gentamicin, and 10 mM HEPES, then transferred to the laboratory for explant culture.

### Myometrial Tissue Culture and Treatments

Approximately 100 mg of tissue from each sample was immediately snap-frozen using liquid nitrogen to serve as a gene expression baseline (0 h). The remaining tissue was dissected into approximately 2 × 2 × 2 mm pieces and washed in serum-free DMEM. To determine treatment effects, myometrial samples were incubated for 48 h in a 37 °C, 95% air/5% CO_2_ humidified incubator in the presence of 5% (v/v) charcoal-stripped, heat-inactivated fetal bovine serum (CSS)-supplemented media (Gibco), P4 (Sigma; 500 nM), estradiol (E2; Sigma; 100 or 400 nM), 8-Bromoadenosine 3′,5′-cyclic monophosphate (8-Br-cAMP; Sigma; 250 µM), phorbol 12-myristate 13-acetate (PMA; Cayman Chemical Company; 0.1 and 1 µM), prostaglandin F_2α_ (PGF_2α_; Cayman Chemical Company; 1, 10, and 100 nM), TSA (Bio-Scientific Pty Ltd; 0.5, 1, 2.5, 5, or 25 µM), SAHA (Cayman Chemical Company; 0.5, 1, 2.5, 5, or 25 µM), or SBHA (Cayman Chemical Company; 0.5, 1, 2.5, 5, or 25 µM). The vehicle was DMSO (0.1% v/v). For the stretch experiments, stainless steel weights were attached to the myometrial tissue strips (2 × 2 × 10 mm) using a nylon thread, and the strips were suspended in 30 mL of culture media in 50 mL tubes as described previously [19, 20]. Media (including treatments) were refreshed after 24 h. After 48 h of incubation, the tissues were snap-frozen using liquid nitrogen and stored at -80 °C for subsequent analyses.

### RNA Extraction, Reverse Transcription, and Real-time Quantitative PCR

RNA was extracted using TRizol Reagent (Thermo Fisher Scientific) according to the manufacturer’s protocol. Homogenization of tissue in TRizol Reagent was performed using a Precellys24 homogenizer (5,000 rpm for 3 × 30 s, with 20 s intervals) (Bertin Instruments). Following extraction, RNA samples were further purified using the TURBO DNA-*free* kit (Thermo Fisher Scientific). RNA concentration (absorbance at 260 and 280 nm) and purity were assessed using a ND-1000 spectrophotometer and RNA integrity was checked by agarose gel electrophoresis before and after DNase treatment. Each RNA sample (0.5 µg of total RNA) was spiked with 0.5 × 10^7^ copies of Alien RNA (Integrated Sciences Pty) and reverse-transcribed using the SuperScript III First-Strand Synthesis System with random hexamer primers (Thermo Fisher Scientific). The Alien RNA transcript is an in vitro transcribed RNA molecule that is non-homologous to any known nucleic acids and, as such, was used as a reference gene for these studies [18–21]. Quantitative RT-PCR was performed using QuantStudio 6 Flex Real-Time PCR (Applied Biosystems). No-reverse transcription negative controls were prepared for each sample. The final volume of each PCR reaction was 20 µL, containing 10 µL of 2 × SYBR Green PCR Master Mix (Thermo Fisher Scientific), master mix cDNA template (corresponding to 10 ng of reverse-transcribed RNA), *AKR1C1* cDNA-specific forward and reverse primers (500 nM). For the reference gene (Alien), the final volume was 20 µL with 1.0 µL of 2.5 µM of Alien Primer Mix (with proprietary sequence), 10 µL of 2 × SYBR Green PCR, the same amount of cDNA as the target genes. No-template control samples were included in each PCR plate to detect any contamination and primer-dimers. The thermal sequence and cycling conditions were as recommended by the manufacturer (Applied Biosystems). Melt curves were determined in each PCR reaction to ascertain the homogeneity of amplified products. *AKR1C1* cDNA primers (Table 1) (Sigma) were designed using Primer-BLAST, optimized, and validated by confirming that single amplicons of appropriate size were generated.

**Table 1:** cDNA primer sequence – *AKR1C1*

Primer	Primer sequence	Amplicon size	GeneBank#
*AKR1C1*	F: AGTTCACCGCTCGCATAA R: GGCTGTAGATAGGCTTAGTGT	60	NM_001353.6

Abbreviation: *AKR1C1* Aldo–keto reductase family 1 member C1

### Protein Extraction, One-dimensional (1D) SDS-PAGE and Immunoblotting

Protein was extracted into sodium dodecyl sulfate (SDS) extraction buffer (2% SDS, 50 mM Tris pH 6.8, 5 mM EDTA) supplemented with PhosSTOP phosphatase inhibitor (Roche) and Complete Mini Protease Inhibitor (Roche). Tissue was homogenized in SDS extraction buffer using a Precellys24 homogenizer (6,500 rpm for 3 × 60 s, with 20 s intervals), after which homogenates were incubated on a rotary mixer for 1 h at 4 °C. Homogenates were then centrifuged at 15,500 g for 15 min at 4 °C (Beckman Coulter Microfuge 20R) and supernatants were collected. Protein concentration was determined using a BCA Protein Assay Kit (Thermo Fisher Scientific).

Myometrial protein extracts (up to 50 µg per lane, due to low AKR1C1 abundance in pregnant human myometrium) were loaded onto 4–12% NuPAGE gels and separated using a Novex Mini-Cell system at constant voltage (200 V for 50 min; Invitrogen). S9 Fraction from Human Liver Extract (Sigma Aldrich, cat# S2442; 0.5 µg/lane) and SDS protein extract from term human placenta (up to 50 µg/lane) were included as positive and negative controls for AKR1C1 detection, respectively. Following 1D SDS-PAGE, proteins were transferred to Hybond-C nitrocellulose (Amersham Biosciences) using the XCell II Blot Module (Invitrogen). Total protein was stained using Ponceau S and imaged using an Amersham Imager 600 (GE Healthcare).

During immunoblotting, all incubations were performed on a rocking platform. Membranes were blocked in 5% skim milk powder in tris-buffered saline (TBS; 500 mM NaCl, 20 mM Tris) for 1 h at room temperature. The blocking solution was decanted and the primary antibody was applied in 5 mL 1% skim milk powder in TBS-Tween-20 (TBS-T; 500 mM NaCl, 20 mM Tris, 0.01% Tween-20) overnight at 4 °C. Antibodies against AKR1C1 (GeneTex, cat# GTX105620, rabbit polyclonal) and a β-actin (Abcam, cat# ab8226, mouse monoclonal) were applied at 1∶1,000 dilution. Blots were subjected to 4 × 5 min washes with 100 mL of TBS-T. Washed blots were then incubated in horseradish peroxidase (HRP)-conjugated anti-rabbit IgG (Cell Signaling Technology, cat# 7074) or anti-mouse IgG (Cell Signaling Technology, cat# 7076) secondary antibody as appropriate, applied at 1∶2500 dilution in 5 mL of 1% skim milk powder in TBS-T for 1 h at room temperature. Blots were washed for 3 × 5 min in 100 mL of TBS-T before immunoreactive products were detected using Immobilon Forte (high sensitivity) Western HRP substrate (Merck Millipore) and visualized using the Amersham Imager 600 (GE Healthcare). To ensure internal controls, blots were first probed with the AKR1C1 antibody before being stripped and re-probed with the antibody against the total proteins. Membranes were stripped by 2 × 5 min incubations in 100 mL of 0.2 M NaOH. Stripped blots were then washed for 3 × 3 min in TBS-T before being re-probed according to the outlined regimen.

### Data and Statistical Analysis

All mRNA abundance data were expressed relative to the Alien reference RNA. The relative mRNA abundance was calculated using the delta Ct (∆Ct) method [22]. Densitometric analysis of immunoreactive protein bands was performed using Amersham Imager 600 analysis software (GE Healthcare). The protein abundance data (optical density (OD; arbitrary units)) were expressed relative to β-actin and for each replicate, relative AKR1C1 protein levels (AKR1C1/β-actin) were normalized to Fresh (0 h) samples. Statistical analyses were conducted with GraphPad Prism software and confirmed using STATA. All mRNA and protein relative abundance values were checked with the Shapiro–Wilk distribution test and not normally distributed data were logarithmically transformed to approach normal distribution. Graphical data are presented as mean ± SEM. For comparison between the two groups, a Student’s *t*-test was used. For comparisons of multiple groups and interactions, analysis of variance and covariance (ANOVA) was performed. *P*-values < 0.05 were considered statistically significant.

## Results

### *AKR1C1* Expression Changes During Ex Vivo Culture

**Fig. 1:**
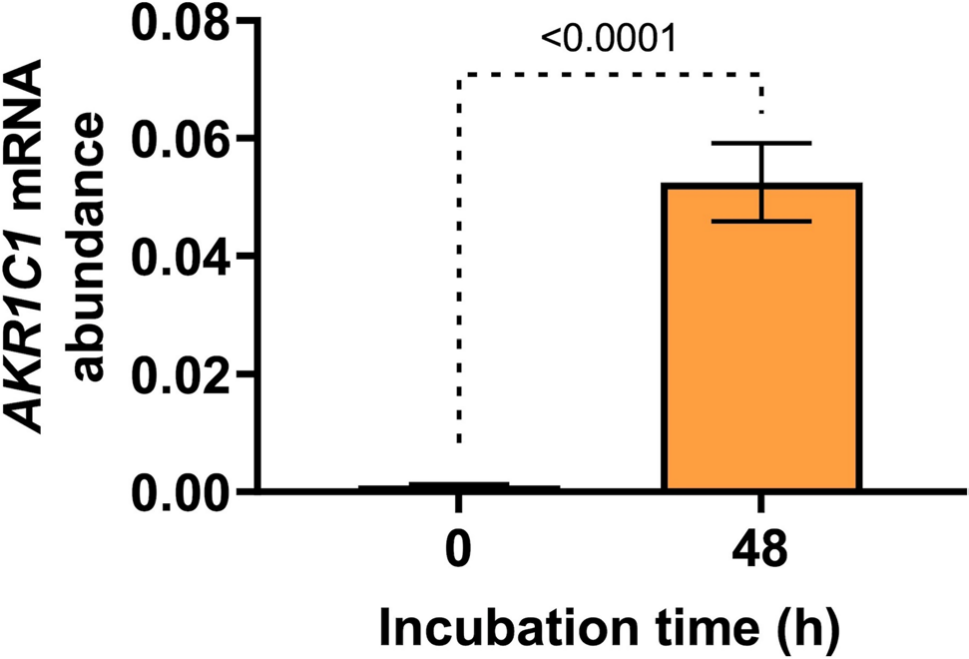
Upregulation of *AKR1C1* expression in term pregnant non-laboring human myometrium cultured for 48 h ex vivo. Relative mRNA abundance of *AKR1C1* was measured either immediately after biopsy (0 h) or following 48 h incubation ex vivo (*n* = 31). *AKR1C1* mRNA abundance is expressed relative to Alien reference. *Data were checked for distribution using the Shapiro–Wilk normality test, logarithmically transformed to approach normal distribution, and analyzed by paired t-test*. *Data are mean* ± *SEM. A significant p-value is indicated*

Myometrial tissues were incubated for 48 h in serum-free media to determine gene expression changes while cultured ex vivo. Consistent with our previous findings of spontaneously upregulated expression of pro-contractile genes [19, 20], myometrial *AKR1C1* mRNA abundance was significantly increased following 48 h incubation (*p* < 0.0001) (Fig. 1).

### Treatment Effects on *AKR1C1* Expression

Considering that the spontaneous upregulation of *AKR1C1* mRNA abundance in culture may model labor-associated changes, we examined whether culture conditions and various treatments can influence the *AKR1C1* expression change ex vivo.

**Fig. 2:**
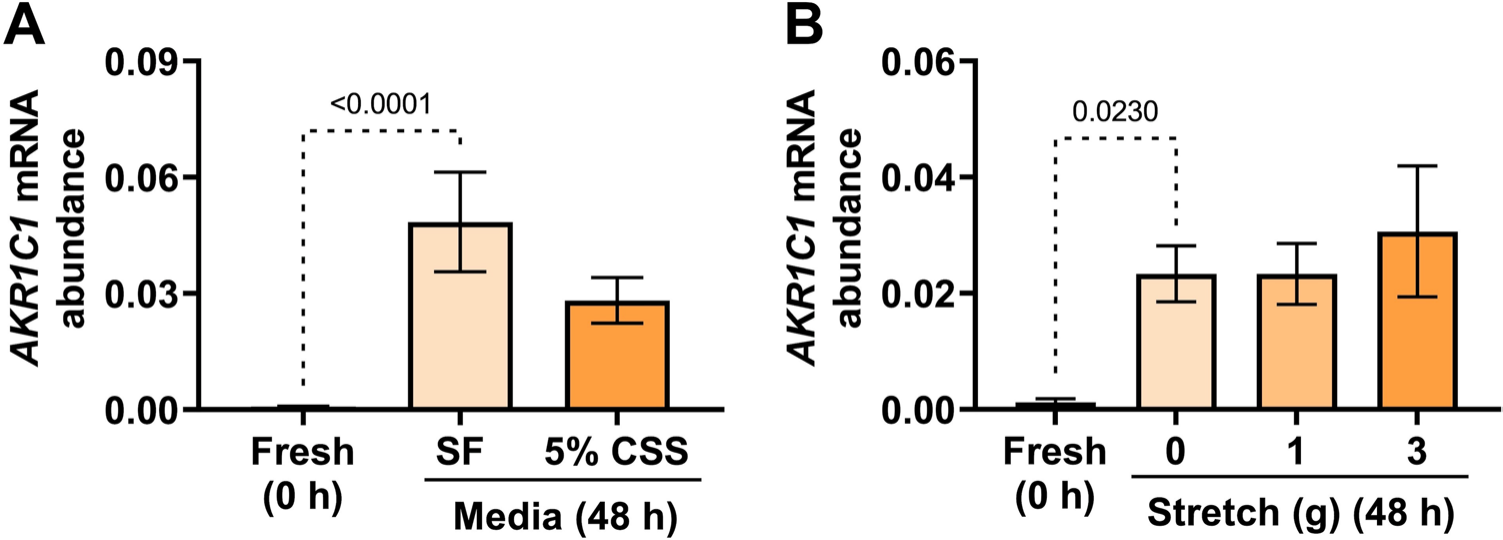
Effect of serum and stretch on the upregulation of myometrial *AKR1C1* expression ex vivo*. AKR1C1* mRNA abundance was measured in (**A**) term not-in-labor myometrial biopsies incubated for 48 h in either serum-free media (*n* = 13) or media supplemented with 5% CSS (*n* = 15), and in (**B**) term non-laboring myometrial strips that were incubated for 48 h while applying 0, 1, and 3 g of stretch (*n* = 3). *AKR1C1* mRNA abundance is expressed relative to Alien reference. *Data were checked for by the Shapiro–Wilk normality test, logarithmically transformed to approach a normal distribution, and then analyzed by 1-way ANOVA with Dunnett’s multiple comparisons test relative to SF (Panel ****A****) and 0 g Stretch (Panel ****B****)*. *Data are mean* ± *SEM. Significant p-values are indicated*

Supplementing the culture media with 5% (v/v) CSS resulted in no significant change in *AKR1C1* mRNA abundance following 48 h incubation compared to serum-free media (*p* = 0.1312) (Fig. 2A). Stretch has been implicated in the regulation of myometrial gene expression during pregnancy [23–26]; however, applying 1 or 3 g of stretch to myometrial tissue strips over 48 h incubation had no effect on the culture-induced change in *AKR1C1* expression, compared to non-stretched (0 g) tissue strips (Fig. 2B).

**Fig. 3:**
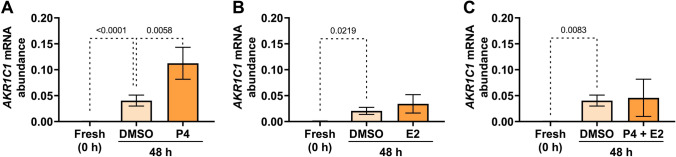
Effect of progesterone (P4) and estradiol (E2) on the upregulation of myometrial *AKR1C1* expression ex vivo*. AKR1C1* mRNA abundance was measured in term non-laboring myometrial explants following 48 h incubation in the presence of (**A**) P4 alone (500 nM) (*n* = 3), (**B**) E2 alone (100 nM) (*n* = 3), or (**C**) P4 (500 nM) + E2 (400 nM) in combination (*n* = 3). *AKR1C1* mRNA abundance is expressed relative to Alien reference. *Data were checked for normality using the Shapiro–Wilk normality test, logarithmically transformed to approach normal distribution, and then analyzed by 1-way ANOVA with multiple comparisons (Dunnett's relative to DMSO in all panels). Data are mean* ± *SEM. Significant p-values are indicated*

Supplementing the media with 500 nM P4 significantly enhanced the culture-induced increase in *AKR1C1* mRNA level compared to vehicle (DMSO)-treated tissues (*p* = 0.0058) (Fig. 3A), whereas supplementing media with E2 alone (100 nM) (*p* = 0.7349) (Fig. 3B) or 500 nM P4 + 400 nM E2 (*p* = 0.7923) in combination had no effect (Fig. 3C).

**Fig. 4:**
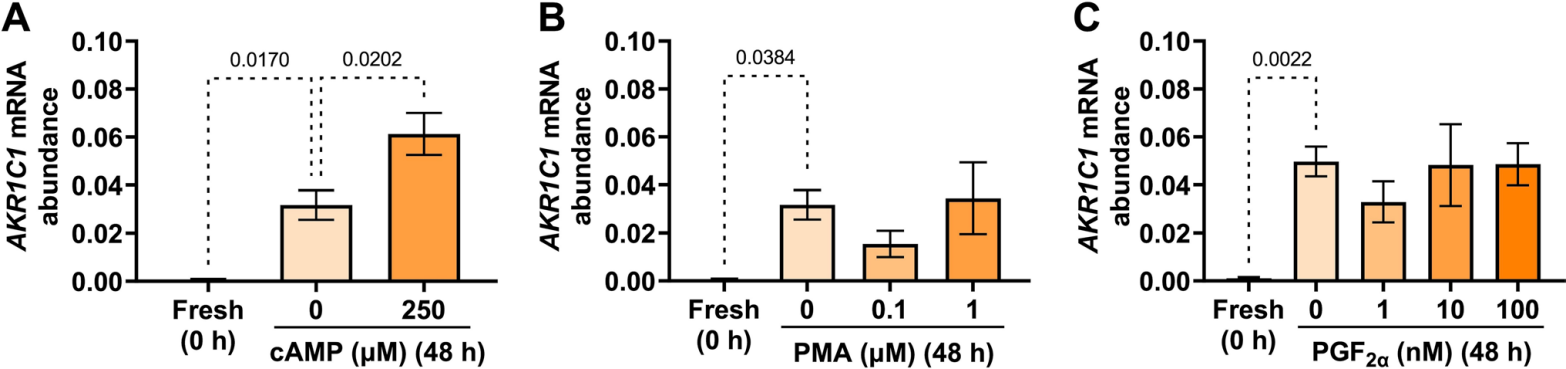
Effect of 8-Br-cAMP (cAMP), phorbol myristate acetate (PMA), and prostaglandin F_2α_ (PGF_2α_) on the upregulation of myometrial *AKR1C1* expression ex vivo*. AKR1C1* mRNA abundance was measured in term non-laboring myometrial explants following 48 h incubation in the presence of (**A**) 8-Br-cAMP (250 µM) (*n* = 3), (**B**) the PKC activator, PMA (0.1, 1.0 µM) (*n* = 3), or (**C**) the prostaglandin, PGF_2α_ (1, 10, 100 nM) (*n* = 3). *AKR1C1* mRNA abundance is expressed relative to Alien reference. *Data were checked for normality using the Shapiro–Wilk normality test and then analyzed by 1-way ANOVA with multiple comparisons (Sidak's relative to 0 in all panels). Data are mean* ± *SEM. Significant p-values are indicated*

Adding 250 µM 8-Br-cAMP to the media significantly enhanced the culture-induced increase in *AKR1C1* mRNA abundance following 48 h incubation, relative to vehicle-treated tissues (*p* = 0.0202) (Fig. 4A), while supplementing media with PMA (0.1 and 1.0 µM) (*p* = 0.3097 and *p* = 0.9884) or PGF_2α_ (1, 10, 100 nM) (*p* = 0.3184, *p* = 0.9997, and *p* = 0.9999) did not significantly enhance or repress the culture-induced increase in *AKR1C1* expression, relative to vehicle controls (Fig. 4B and 4C, respectively).

**Fig. 5:**
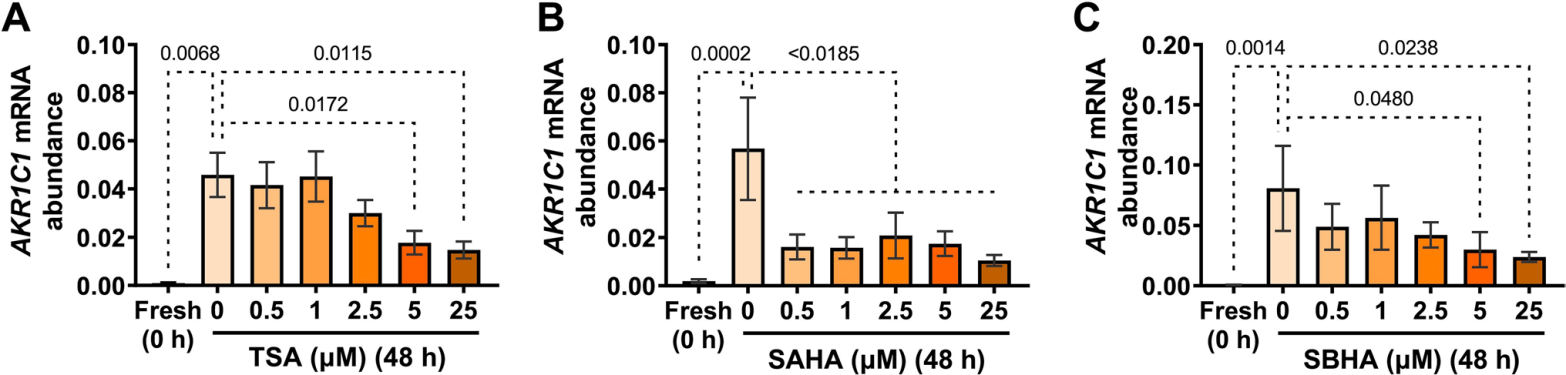
Effect of histone deacetylase inhibitors (TSA, SAHA, and SBHA) on the upregulation of myometrial *AKR1C1* expression ex vivo*. AKR1C1* mRNA abundance was measured in term non-laboring myometrial explants following 48 h incubation in the presence of (**A**) TSA (0.5, 1, 2.5, 5, 25 µM) (*n* = 8), (**B**) SAHA (0.5, 1, 2.5, 5, 25 µM) (*n* = 5) or (**C**) SBHA (0.5, 1, 2.5, 5, 25 µM) (*n* = 4). *AKR1C1* mRNA abundance is expressed relative to Alien reference. *Data were checked using the Shapiro–Wilk normality test and analyzed by 1-way ANOVA with multiple comparisons (Dunnett's relative to 0). Data are mean* ± *SEM. Significant p-values are indicated*

Supplementing media with TSA, SAHA, or SBHA (0.5, 1, 2.5, 5, 25 µM) repressed the culture-induced increase in *AKR1C1* mRNA abundance following 48 h incubation. Relative to vehicle (DMSO)-treated controls, TSA significantly repressed *AKR1C1* upregulation at 5 (*p* = 0.0172) and 25 µM (*p* = 0.0115) (Fig. 5A). Consistent results were observed for SBHA, which also significantly repressed *AKR1C1* upregulation at 5 (*p* = 0.0480) and 25 µM (*p* = 0.0238) (Fig. 5C), whereas SAHA significantly repressed *AKR1C1* upregulation, compared to vehicle treatment, at 0.5 (*p* = 0.0070), 1 (*p* = 0.0045), 2.5 (*p* = 0.0181), 5 (*p* = 0.0066) and 25 µM (*p* = 0.0014) (Fig. 5B).

#### AKR1C1 Protein Levels

**Fig. 6:**
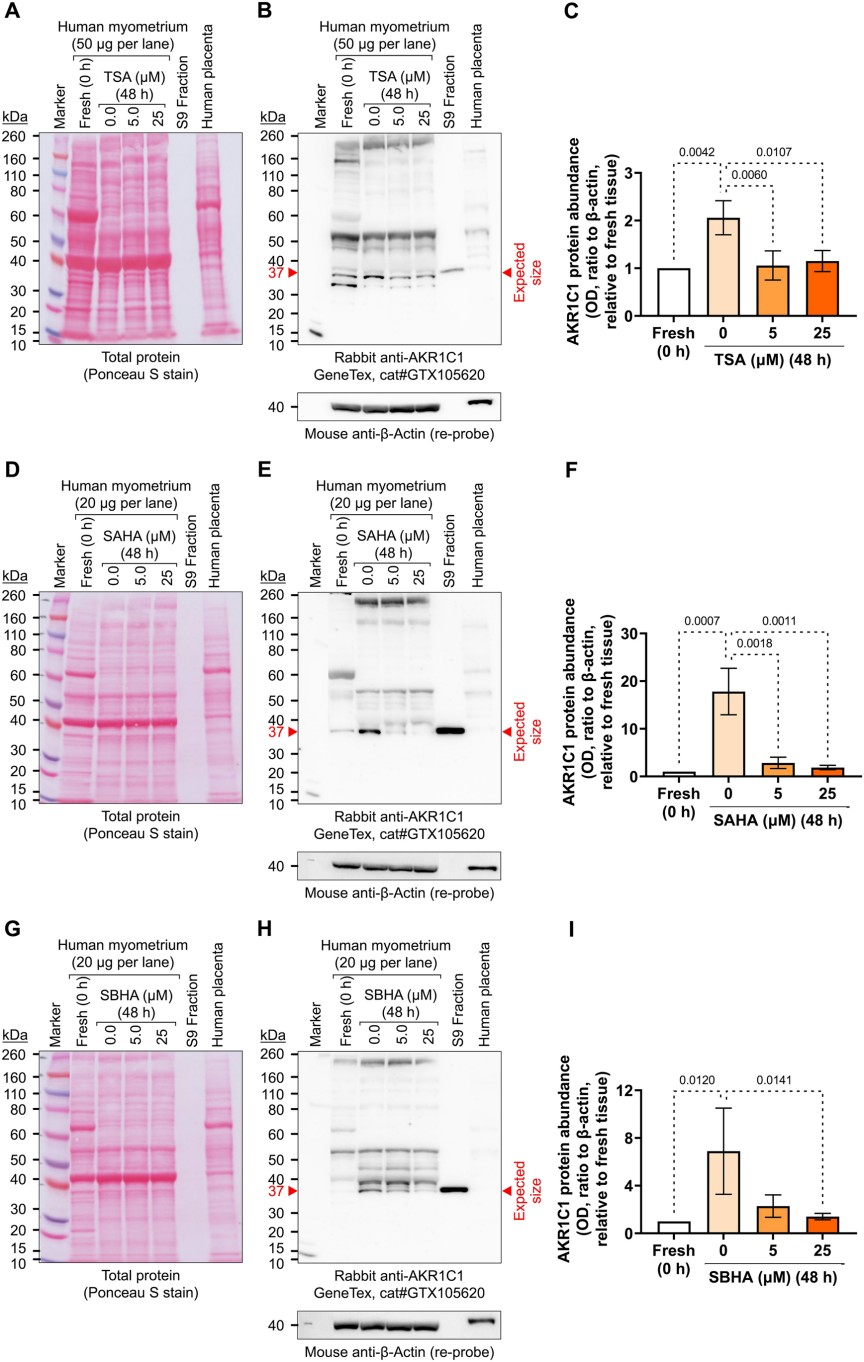
Effect of TSA, SAHA, and SBHA on the upregulation of myometrial AKR1C1 protein abundance ex vivo*.* Protein extracts from fresh (0 h) term non-laboring myometrium or myometrial explants incubated for 48 h in the presence of 0, 5, or 25 µM TSA (*n* = 4) (panels **A** – **C**), SAHA (*n* = 5) (panels **D** – **F**), or SBHA (*n* = 3) (panels G – I) were separated by SDS-PAGE (20—50 µg/lane) and then transferred to a nitrocellulose membrane. S9 Fraction from human liver extract (0.5 µg/lane) and human placenta extract (20—50 µg/lane) were included as positive and negative controls, respectively. Total protein transfer was visualized by Ponceau S staining and then imaged (panels **A**, **D**, **G**). Membranes were then probed for AKR1C1 detection using GeneTex (cat# GTX105620) rabbit anti-AKR1C1 polyclonal antibody (1:1000) and anti-rabbit-HRP secondary antibody (1:2000). Representative images show immunoreactive bands detected using Immobilon Forte chemiluminescence reagent (panels **B**, **E**, **H**). Blots were then stripped and re-probed using mouse anti-β-actin (1:1000) and anti-mouse-HRP (1:2000). The molecular weight marker was Novex™ Sharp Pre-stained Protein Standard. The optical density of the dominant immunoreactive band corresponding to the expected molecular weight of AKR1C1/20α-HSD (37 kDa) was measured and protein abundance expressed relative to β-actin and normalized to fresh (0 h) myometrial explants (panels **C**, **F**, **I**). *Data were checked using the Shapiro–Wilk normality test and analyzed by 1-way ANOVA with multiple comparisons (Dunnett’s relative to 0). Data are mean* ± *SEM. Significant p-values are indicated*

Western blotting of pregnant human myometrial protein extracts revealed that the GeneTex anti-AKR1C1 antibody was not specific for AKR1C1 in that the antibody cross-reacted with a triplicate of protein bands in the vicinity of the expected molecular weight of AKR1C1 (37 kDa) (Fig. 6). The dominant (middle) band out of the three was further analyzed; however, it remains to be confirmed whether the dominant band is indeed AKR1C1. Nonetheless, the analysis revealed that the intensity of the dominant protein band significantly increased following 48 h incubation (*p* = 0.0042 in Fig. 6C, *p* = 0.0007 in Fig. 6F, and *p* = 0.0120 in Fig. 6I). Supplementing media with TSA at 5 (*p* = 0.0060) and 25 µM (*p* = 0.0107) repressed this culture-induced increase of band intensity (*n* = 4) (Fig. 6C). Treatment with SAHA also repressed the cultured-induced increase of protein band intensity at both 5 (*p* = 0.0018) and 25 µM (*p* = 0.0011) (*n* = 5) (Fig. 6F), while SBHA significantly repressed the culture-induced increase of the protein band at 25 µM (*p* = 0.0141) (*n* = 3) (Fig. 6I).

## Discussion

Prior studies have shown that *AKR1C1* mRNA abundance and 20α-HSD protein levels increase with labor onset in pregnant human myometrium at term [14, 16, 17]. Recently, we reported an average sixfold increase in myometrial *AKR1C1* mRNA abundance in association with labor onset at term [18]. Whilst the regulation of *AKR1C1* has been studied in human telomerase-immortalized myometrial (hTERT-HM) cells [17], it remains crucial to examine *AKR1C1* regulation in human myometrial tissue in short-term cultures reflecting the in vivo state more closely. Accordingly, we utilized our ex vivo labor model in which key labor-associated genes (*ESR1*, *PTGS2, OXTR,* and *PGR*) undergo culture-induced expression changes within 48 h that are consistent with transitioning to a labor-like phenotype [19, 20]. Within this model, we observed a significant increase in *AKR1C1* mRNA abundance across the 48 h culture period (Fig. 1). This increase reflects the documented upregulation of *AKR1C1* expression that occurs in association with term labor [18] and further supports that non-laboring myometrium transitions toward a labor-like phenotype during ex vivo culture. The model enabled us to assess whether treatments known to influence myometrial activity affected the culture-induced increase in *AKR1C1* expression and contribute to enhancing or repressing the ex vivo transition toward a labor-like phenotype.

Upon examining the effects of various pro-quiescence and pro-contractile agents, we found that treatment with P4 significantly upregulated *AKR1C1* expression beyond the culture-induced change (Fig. 3A). The possibility that P4 can upregulate its own metabolism and exert negative feedback on target tissue P4 sensitivity is intriguing, as P4 can be anticipated intuitively to repress myometrial *AKR1C1* expression for maintaining high P4 levels throughout pregnancy. Clearly, E2 blocks this effect. One may also consider that *AKR1C1* and its paralogues have loose steroid selectivity and can be involved in the reductive metabolism of intrauterine androgens and estradiol, in addition to P4 [27, 28]. To assume that the sole function of *AKR1C1* is to control P4 levels is a simplification, and further studies are required to precisely delineate the role AKR1C family members have in shaping the steroid environment of the myometrium.

*AKR1C1* mRNA expression was also upregulated by cAMP (Fig. 4A), which is a powerful suppressor of myometrial contractions. However, genomic cAMP signaling has been shown to exert both pro-relaxatory and pro-contractile roles [29, 30] in that prolonged cAMP exposure can activate pro-contractile mitogen-activated protein kinase (MAPK) signaling [31] and upregulate pro-contractile genes (reviewed by Butler et al. [30]). It is plausible that cAMP may drive *AKR1C1* expression in pregnant human myometrium as part of a pro-contractile action. More in-depth analyses, such as time-dependency studies, are required to determine whether cAMP exerts differential effects on *AKR1C1* expression following short- or prolonged exposure and determine the underlying molecular mechanism(s).

Recently, Nadeem et al. [17] reported that treating hTERT-HM cells with the pro-inflammatory mediator TPA, also known as PMA (20 ng/mL; 32.4 nM), induced *AKR1C1* expression and increased AKR1C1 protein levels. At odds with this report, we observed no effect of TPA/PMA (0.1 and 1 µM) on myometrial *AKR1C1* expression (Fig. 4B). The difference may be attributable to the different model systems used (immortalized myometrial cell line *vs* primary myometrial tissue) or the different TPA/PMA concentrations applied. The concentrations of PMA used in this study (0.1 and 1 µM) were effective in our former study at significantly increasing the *PR-A/PR-B* ratio in human myometrial explants ex vivo*,* compared to 48 h vehicle-treated tissue [20], but in this study, the same PMA concentrations did not affect *AKR1C1* expression.

Within our ex vivo labor model, TSA, SAHA, and SBHA each dose-dependently inhibited the culture-induced increase in *AKR1C1* expression (Fig. 5) and significantly repressed upregulation of the dominant immunoreactive protein band detected at the expected molecular weight for AKR1C1 (Fig. 6). This is consistent with a pro-relaxation/pro-quiescence effect, as repressing *AKR1C1* expression and 20α-HSD levels are anticipated to contribute toward preventing P4 metabolism in the myometrium, thus promoting the maintenance of P4 signaling through PR-B. The precise mechanism by which HDACis inhibit *AKR1C1* expression is yet to be determined; however, it may be through HDACis affecting the acetylation of transcription factors that regulate *AKR1C1* expression or by effects on chromatin structure (nucleosome acetylation) within gene regulatory regions [32].

Previously, we have demonstrated that TSA exerts a pro-quiescence effect on pregnant human myometrium by repressing upregulation of *PR-A*, but not *PR-B*, to maintain a low *PR-A*/*PR-B* ratio. Our team was the first to suggest that the “*efficacy of progesterone administration may be enhanced if an agent such as TSA could be administered to preserve or even restore progesterone sensitivity in women with threatened preterm labor*” [20]. Here, our data demonstrating that HDACis exert an additional pro-quiescence effect of repressing *AKR1C1* upregulation in pregnant human myometrium further supports the possibility that co-administration of a HDACi may enhance the effectiveness of P4 therapy. In agreement with this, Zierden et al. [33] showed that administration of a TSA nanosuspension in combination with a nanosuspension of P4 was effective at significantly reducing the rate of inflammation-induced preterm birth in mice, whereas the nanosuspension of P4 alone had no significant effect. Moreover, the TSA + P4 combination therapy led to the birth of neurotypical offspring [33], which is consistent with TSA having been shown to extend mouse pregnancy with no obvious impacts on litter size or fetal viability [34]. New developments in drug delivery techniques, such as mucus penetrating nanoparticles [33, 35] and uterine-targeted nanoliposomes [36], may therefore allow the selective deployment of HDACis to the myometrium for maintaining P4 sensitivity and achieving tocolysis while reducing the likelihood of maternal or fetal side-effects. This work, showing that HDACis have the pro-relaxatory effect of suppressing myometrial *AKR1C1* upregulation ex vivo, adds to prior work showing that TSA regulates the *PR-A/PR-B* ratio [20], and that vaginal administration of P4 combined with TSA can prevent preterm birth in mice [33]. Further research will elucidate the molecular mechanisms by which HDACis regulate the genes controlling P4 action in the uterus, which may include affecting the acetylation of transcription factors that regulate these genes, or altering histone acetylation, thus changing chromatin accessibility at gene regulatory regions in the myometrium [33].

A strength of this study is that we have used an ‘ex vivo labor’ model of the human myometrium, which is uniquely informative about the responses of the tissue to treatments that influence the labor-associated uterine phenotype transition. A caveat is the limited reliability of our protein data. Extensive efforts were made to evaluate AKR1C1 protein levels in extracts of pregnant human myometrium via Western blotting; however, this proved difficult due to the available anti-AKR1C1 antibodies cross-reacting with a triplicate of protein bands within the vicinity of the expected molecular weight for human AKR1C1. It is possible that the triplicate bands correspond to AKR1C1 (37 kDa), AKR1C2 (36 kDa), and AKR1C3 (34 kDa), given that in humans, AKR1C1 shares 97.8% and 87.9% sequence homology with AKR1C2 and AKR1C3, respectively. The lack of reliable antibodies for distinguishing between aldo–keto reductase family members has been noted by others [37], and due to this limitation, the precise identity of the triplicate gene products remains currently unknown [18]. A further limitation is that we did not assess the downstream effects of AKR1C1 expression changes, including P4 metabolic activity, AKR1C1 enzyme activity, and canonical P4-responsive marker gene products (e.g., *FKBP5* mRNA). A full characterization of the ex vivo model will include these aspects as well as the contribution of other steroid metabolic enzymes in the various myometrial tissue compartments potentially affecting local P4 availability. We also note that myometrial tissue is heterogeneous, meaning that low levels of non-myometrial cell types may be present within the explants, and the presence of these cells has the potential to impact both the mRNA and protein data. Nonetheless, it is relevant to consider that P4, being a steroid, may diffuse within the tissue, and AKR1C1 in any tissue component may affect P4 availability locally.

In summary, we have demonstrated that myometrial *AKR1C1* mRNA expression, and the dominant immunoreactive band representing AKR1C1/20α-HSD protein abundance, significantly increased in the term, not-in-labor pregnant human myometrium cultured ex vivo; which is consistent with the transition to a pro-labor phenotype and the upregulation of other labor-associated genes in our ex vivo labor model. The culture-induced increase in *AKR1C1* expression was significantly exacerbated by P4 and cAMP, while the HDACis, TSA, SAHA, and SBHA, dose-dependently repressed the ex vivo upregulation of myometrial *AKR1C1* mRNA and AKR1C1/20α-HSD protein levels, indicating that protein acetylation plays a role in myometrial *AKR1C1* expression control. Moreover, these findings indicate that suppression of AKR1C1/20α-HSD mediates, in part, the pro-relaxation effects of HDACis regulating P4 sensitivity in human myometrium, complementing the previously described ability of these drugs to selectively repress labor-promoting *PR-A* expression in this tissue. Our data further highlight the potential of HDACi compounds to be clinically useful for pregnancy maintenance in a setting where they can be safely deployed.

## Data Availability

Data available on request due to privacy/ethical restrictions.

## Glossary of Terms

*1D*: One-dimensional
*20α-HSD*: 20α-Hydroxysteroid dehydrogenase
*20α-OHP*: 20α-Hydroxyprogesterone
*8-Br-cAMP*: 8-Bromoadenosine 3′,5′-cyclic monophosphate
*AKR*: Aldo-keto reductase
*AKR1C1*: Aldo-keto reductase family 1 member C1 gene
*AKR1C2*: Aldo-keto reductase family 1 member C2 gene
*AKR1C3*: Aldo-keto reductase family 1 member C3 gene
*ANOVA*: Analysis of variance
*BCA*: Bicinchoninic acid
*BMI*: Body mass index
*cAMP*: Cyclic adenosine monophosphate
*CAP*: Contraction-associated protein
*CS*: Cesarean section
*CSS*: Charcoal-stripped fetal bovine serum
*Cx43*: Connexin 43
*EDTA*: Ethylenediaminetetraacetic acid
*DME*: Dulbecco’s modified eagle medium
*E2*: Estradiol
*GJA1*: Gap junction alpha-1 protein
*HDAC*: Histone deacetylase
*HDACi*: Histone deacetylase inhibitor
*HRP*: Horseradish peroxidase
*hTERT-HM*: Human telomerase immortalized myometrial cells
*LPS*: Lipopolysaccharide
*MAPK*: Mitogen-activated protein kinase
*mRNA*: Messenger ribonucleic acid
*NF-κB*: Nuclear factor kappa-light-chain-enhancer of activated B cells
*OXTR*: Oxytocin receptor gene
*P4*: Progesterone
*PBS*: Phosphate-buffered solution
*PGF*_*2α*_: Prostaglandin F_2α_
*PMA*: Phorbol myristate acetate
*PR*: Progesterone receptor
*RT-PCR*: Real-time quantitative polymerase chain reaction
*PTGS2*: Prostaglandin-endoperoxide synthase 2 gene
*RNA*: Ribonucleic acid
*SAHA*: Suberoylanilide hydroxamic acid
*SBHA*: Suberoyl bis-hydroxamic acid
*SDS*: Sodium dodecyl sulfate
*SDS-PAGE*: Sodium dodecyl sulfate–polyacrylamide gel electrophoresis
*TBS*: Tris buffered saline
*TBS-T*: Tris buffered saline tween-20
*TPA*: 12-O-Tetradecanoylphorbol-13-acetate
*TSA*: Trichostatin A
